# Vibrations and Spatial Patterns Change Effective Wetting Properties of Superhydrophobic and Regular Membranes

**DOI:** 10.3390/biomimetics1010004

**Published:** 2016-08-04

**Authors:** Rahul Ramachandran, Michael Nosonovsky

**Affiliations:** Mechanical Engineering Department, University of Wisconsin-Milwaukee, 3200 N Cramer St., Milwaukee, WI 53211, USA; ramacha6@uwm.edu

**Keywords:** vibrations, micro/nanotopography, wetting, membrane, oil-water separation

## Abstract

Small-amplitude fast vibrations and small surface micropatterns affect properties of various systems involving wetting, such as superhydrophobic surfaces and membranes. We review a mathematical method of averaging the effect of small spatial and temporal patterns. For small fast vibrations, this method is known as the method of separation of motions. The vibrations are substituted by effective force or energy terms, leading to vibration-induced phase control. A similar averaging method can be applied to surface micropatterns leading surface texture-induced phase control. We argue that the method provides a framework that allows studying such effects typical to biomimetic surfaces, such as superhydrophobicity, membrane penetration and others. Patterns and vibration can effectively jam holes and pores in vessels with liquid, separate multi-phase flow, change membrane properties, result in propulsion, and lead to many other multiscale, non-linear effects. Here, we discuss the potential application of these effects to novel superhydrophobic membranes.

## 1. Introduction

Biomimetic functional surfaces find many applications, including various types of membranes for water filtration. Recent advances in nano/microtechnology have made it possible to design biomimetic functional surfaces with micro/nanotopography with various properties, such as non-adhesion, the ability for liquid manipulation at the microscale, and other applications. In order to understand the structure-property relationships in these novel materials and surfaces, it is important to study how micro/nanotopography changes surface properties resulting in effective macroscale properties. While micro/nanotopography usually constitutes a number of periodic spatial patterns, there is a similarity between the effect of small-scale patterns and small-amplitude fast vibrations.

**Figure 1:**
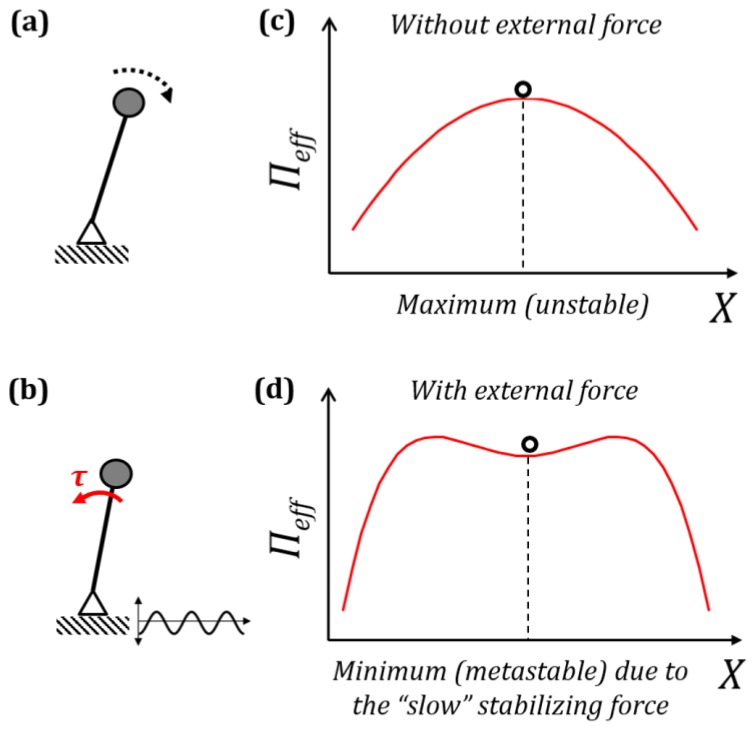
Separation of motions. (**a**) Unstable inverted pendulum; (**b**) Stabilized inverted pendulum on a vibrating foundation; (**c**) Energy profile without external force; (**d**) Energy profile with external force.

Using the mathematical method of separation of motions, small fast vibrations can be substituted by an effective force perceived at the larger scale. The simplest mechanical example of this effect is the vibration-induced stabilization of the inverted pendulum on a vibrating foundation often called the Kapitza pendulum [1,2,3]. The upside down position of the pendulum is unstable (Figure 1a). However, if the foundation vibrates with a small amplitude and high frequency—relative to the size and natural frequency of the pendulum, respectively—, the inverted equilibrium position can become stable (Figure 1b). This is perceived, at the macroscale, as an effective stabilizing “spring force” which maintains the pendulum in equilibrium. In other words, small fast vibrations can be substituted by an effective force, which stabilizes the inverted pendulum. This effect of the vibration-induced stabilization can be extended to the case of a double and multiple pendulums [4] and even of “effective hardening” a flexible stiff rope (i.e., a flexible rope which still possesses some stiffness akin to a stiff beam) [5].

The effect can be extended to a broad range of mechanical systems including those involving the fluid flow and propulsion [6], as well as systems that are not purely mechanical. Thus, small fast vibrations can cause shear thickening of non-Newtonian fluids, which is perceived as an effective force acting upon the liquid leading, for example, to the rise of the figurines in the cornstarch—sometimes called the “cornstarch monster” trick. Beyond that, liquid droplets can bounce indefinitely in a non-coalescing state above a vibrating bath of bulk liquid, which is perceived as a “vibro-levitation” effect [7]. Furthermore, small fast vibrations can lead to the vibration-induced effective phase transitions, for example, when a granular material flows through a hole in a vessel like a liquid, or when vibrations of a vessel with a liquid jams the hole and prevents liquid penetration.

Blekhman [8] suggested that the stability problem of an inverted pendulum on a vibrating foundation has relevance to a diverse class of non-linear effects involving dynamic stabilization of statically unstable systems ranging from the vibrational stabilization of beams, to the transport and separation of granular material, soft matter, bubbles and droplets, as well as the synchronization of rotating machinery. In these problems, the small fast vibrational motion can be excluded from the consideration and substituted by effective slow forces acting on the system causing the stabilizing effect. It has been suggested that the “effective hardening” of fibers in a composite material can lead to a novel class of “dynamic materials” with effective properties controlled by an externally applied electric field [9].

Surface micro/nanotopography can also change effective material or surface properties. The micro/nanotopography can be thought of as a spatial pattern while small fast vibrations constitute periodic temporal patterns. For example, properly controlled micro/nanotopography can affect the wettability of surfaces as seen in the case of superhydrophobic [10] and non-adhesive surfaces [11], icephobicity [12], liquid flow [6] and filtration [13]. The novel field of texture-induced phase transition has recently emerged from the area of the superhydrophobicity [14].

Both with the spatial and temporal modulations, a small-scale phenomenon, such as the micro/nanotopography or vibrations, can be effectively substituted by an effect perceived at the larger scale, such as the stabilizing or “vibro-levitation” force [15].

In our earlier publication, we discussed how small patterns can be used for liquid flow, including the shark-skin effect and blood flow applications (“haemeophobic” or blot-clotting preventing surfaces) [6]. In this review, we focus on how small fast vibrations and micro/nanotopography can affect surface physicochemical properties, with emphasis on superhydrophobic and regular membranes. We will introduce the mathematical method of separation of motions. The stabilization of the inverted pendulum is the model example for the application of this method. We then discuss the analogy between vibrations and spatial patterns. This will be followed by a discussion of the effect of topography and vibrations on membrane permeability.

## 2. Effect of Small Fast Vibrations and Surface Patterns

In this section we introduce the method of separation of motions and apply this method to determine the stability criteria for the inverted pendulum and numerous similar systems, both in mechanics and physical chemistry. Then we discuss the mathematical analogies, which correlate with vibrations and spatial patterns.

### 2.1. Separation of Motion and Effective Forces

The method of separation of motions was first suggested by the Russian physicist Kapitza in 1951 [3] to study the stability of a pendulum on a vibrating foundation. The method was generalized for the case of an arbitrary motion in a rapidly oscillating field and it is discussed in the classical textbook on theoretical physics by Landau and Lifshitz [16].

Consider a point mass *m* in an oscillatory potential field ІІ(*x*), where *x* is spatial coordinate, with the minimum of the potential energy corresponding to the stable equilibrium. The force acting on the mass is given by −dΠ/dx, therefore, the equation of motion of the system is $m\ddot{x}=-d\Pi /dx$. In addition, to the time-independent potential field Π(x), a “fast” external periodic force fcosΩt acts upon the mass with a small amplitude *f* and high frequency $\Omega >>\sqrt{\left(d^{2}\Pi /dx^{2}\right)/m}$, which is much higher than natural frequency. The equation of motion then becomes

(1) $$m\ddot{x}=-\left(d\Pi /dx\right)+f\cos\Omega t$$

The motion of the mass can be represented as a sum of the “slow” oscillations X(t) due to the “slow” force −dΠ/dx and small “fast” oscillations ξ(t) due to the “fast” force fcosΩt,

(2) x(t)=X(t)+ξ(t)

The mean value $\bar{\xi }\left(t\right)$ of this fast oscillation over its period 2π/Ω is zero, whereas X(t) changes only slightly during the same period.

(3) $$\bar{\xi }\left(t\right)=\frac{\Omega }{2\pi }\int_{0}^{2\pi /\Omega }\xi \left(t\right)dt=0$$

(4) $$\bar{X}\left(t\right)\approx X\left(t\right)$$

Therefore, the mean location of the mass can be written as:

(5) $$\bar{x}\left(t\right)=\bar{X}\left(t\right)+\bar{\xi }\left(t\right)\approx X\left(t\right)$$

and the mean value of the second derivative:

(6) $$\ddot{\bar{x}}\left(t\right)\approx \ddot{X}\left(t\right)$$

In Equations (2)–(6) quantities with a bar are averaged quantities over the period of 2π/Ω. Substituting Equation (2) into Equation (1) and using the Taylor series first-order terms in powers of ξ:

(7) $$m\ddot{X}+m\ddot{\xi }=-\frac{d\Pi }{dx}-\xi \frac{d^{2}\Pi }{dx^{2}}+f\cos\Omega t+\xi \frac{\partial \left(f\cos\Omega t\right)}{\partial X}$$

The slow and fast terms in Equation (7) must separately be equal. The second derivative of small fast oscillations $\ddot{\xi }$ is proportional to $\Omega^{2}$, which is a large term. On the other hand, the terms on the right-hand side of Equation (7), containing the small ξ, can be neglected. The term −dΠ/dx is a slow restoring force. The remaining fast terms can be equated, $m\ddot{\xi }=f\cos\Omega t$. Integrating this equation with respect to time *t*,

(8) $$\xi =-\frac{f\cos\Omega t}{m\Omega^{2}}$$

Averaging Equation (7) with respect to time, substituting the relation $\frac{\Omega }{2\pi }\int_{0}^{2\pi /\Omega }f\cos\Omega tdt=0$ and combining Equations (3)–(6), and Equation (8) gives:

(9) $$m\ddot{X}=-\frac{d\Pi }{dX}+\bar{\xi \frac{\partial \left(f\cos\Omega t\right)}{\partial X}}=-\frac{d\Pi }{dX}-\frac{1}{m\Omega^{2}}\bar{f\cos\Omega t\frac{\partial \left(f\cos\Omega t\right)}{\partial X}}$$

$$m\ddot{X}=-\frac{d\Pi }{dX}-\frac{1}{2m\Omega^{2}}\bar{\frac{\partial {\left(f\cos\Omega t\right)}^{2}}{\partial X}}$$

This can be written as $m\ddot{X}=-\frac{d\Pi_{eff}}{dX}$ where $\Pi_{eff}$ is an effective potential energy given by:

(10) $$\Pi_{eff}=\Pi +\frac{1}{2m\Omega^{2}}\frac{\Omega }{2\pi }\int_{0}^{2\pi /\Omega }{\left(f\cos\Omega t\right)}^{2}dt=\Pi +\frac{f^{2}}{4m\Omega^{2}}=\Pi +\frac{m}{2}{\bar{\dot{\xi }}}^{2}$$

Thus, the effect of fast vibrations ξ when averaged over the time period 2π/Ω is equivalent to the additional term $m{\bar{\dot{\xi }}}^{2}/2$ on the right-hand side in Equation (10). This term is the mean kinetic energy of the system under fast oscillations. Thus, small fast vibrations can be substituted by an additional term in the potential energy resulting in the same effect oscillations have on the system. The most interesting case is when this term affects the state of the equilibrium of a system. Let us say, in the absence of vibrations a system has an effective potential energy $\Pi_{eff}=\Pi$ with a local maximum of the potential energy (Figure 1c). Vibrations can bring this system to a stable equilibrium due to the additional term discussed before, creating a local minimum of the potential energy (Figure 1d). In such cases, the small fast vibrations have a stabilizing effect on the state of equilibrium.

Blekhman [8] has applied the method of separation of motions to many mechanical systems and suggested what he called the “vibrational mechanics” as a tool to describe diverse effects in the mechanics of solid and liquid media, from effective “liquefying” of the granular media, which can flow through a hole like a liquid when on a vibrating foundation, to the opposite effect of “solidifying” liquid by jamming a hole in a vessel on a vibrating foundation, as well as the vibro-synchronization of the phase of two rotating shafts on a vibrating foundation.

Blekhman [8] has also suggested an elegant interpretation of the separation of motions. According to his interpretation, there are two different observers who can look at the vibrating system. One is an ordinary observer in an inertial frame of reference in which one can see both small, *ζ*, and large, *X*, oscillations. The other one is a “special” observer in a vibrating frame of reference, which does not see the small-scale motion ξ, possibly, due to a stroboscopic effect or because one’s vision is not sensitive enough to see the small-scale motion. As a result, what is seen for the ordinary observer as an effect of the fast small vibrations is perceived by the special observer as an effect of some new effective force. This fictitious force is similar to the inertia force, which is observed in non-inertial frames of reference. Furthermore, when the stabilizing effect occurs, the special observer attributes the change in effective potential energy to fictitious slow stabilizing forces or moments. The additional slow stabilizing force for the system (or torque for rotational systems) *V* can be written as:

(11) $$V=-\frac{\partial }{\partial X}\left(\frac{1}{2m\Omega^{2}}\frac{\Omega }{2\pi }\int_{0}^{2\pi /\Omega }{\left(f\cos\Omega t\right)}^{2}dt\right)=-\frac{\partial }{\partial X}\left(\frac{f^{2}}{4m\Omega^{2}}\right)$$

For an inverted pendulum on a harmonically vibrating foundation (AcosΩt), where *A* is the amplitude and Ω is the frequency, the equation of motion can be written in terms of its angular displacement ψ as:

(12) $$L\ddot{\psi }=g\sin\psi -A\Omega^{2}\sin\psi \cos\Omega t$$

The inverted position can become stable if the condition $A^{2}\Omega^{2}>2gL$ holds (Figure 1b). Using Equations (11) and (12) the effective stabilizing torque can be obtained [7,15] as:

(13) $$\tau =-\frac{mA^{2}\Omega^{2}}{4}\sin2\psi$$

which, in the small angle approximation sin2ψ≈2ψ is also equivalent to the inverted pendulum being stabilized by a spring with the torsional spring constant $k=\frac{mA^{2}\Omega^{2}}{2}$. Similarly, this method can be used to derive the effective stabilizing torques on inverted multiple pendulums on a vibrating foundation, and the increase in stiffness of a vibrating flexible rope to prevent buckling—the “Indian rope trick” [15]. Thus, small fast vibrations can affect the equilibrium and manifest as an effective stabilizing force.

The effective stabilizing force in Equation (11) was obtained as an average over time. In the following sections, we use similar averaging over temporal or spatial variable to study the effect of temporal or spatial patterns on physicochemical properties.

### 2.2. Kirchhoff’s Analogy between Spatial and Temporal Patterns

**Figure 2:**
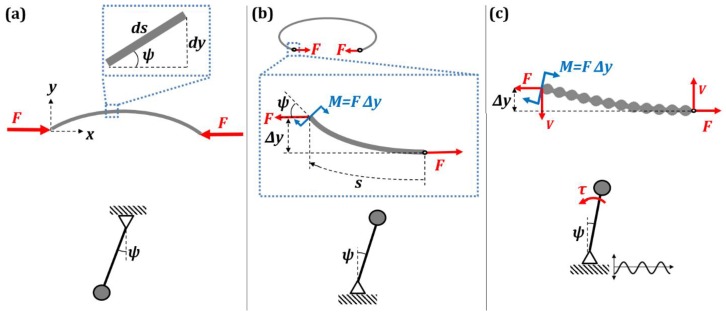
Similarity of bending of a beam and vibration of a pendulum. The deflection of a beam due to (**a**) compressive load *F* (corresponding to the stable equilibrium of a regular pendulum) and (**b**) tensile load *F* (corresponding to the unstable equilibrium of an inverted pendulum); (**c**) The waviness of the beam would stabilize the equilibrium similarly to the vibrations stabilizing an inverted pendulum [6].

Similar to how small vibrations can be substituted by an effective force, small-amplitude patterns in space can have the same effect. The so-called Kirchhoff’s dynamical analogy establishes similarity between the static bending shape of a beam and the dynamics of motion of a rigid body [17,18]. Let us consider a slender beam of area moment of inertia *I*, and modulus of elasticity *E*, whose end points are loaded by an axial compressive force *F* as shown in Figure 2a. The slope at any point (*x*, *y*) is denoted by the angle ψ. For any small element *ds* on the beam dy/ds=sinψ. Bending moment at (*x*, *y*) is given by EIdψds=−Fy. By combining these equations, we get a differential equation, which describes the spatial bending patterns on the beam [19].

(14) $$\frac{d^{2}\psi }{ds^{2}}+\frac{F}{EI}\sin\psi =0$$

This is similar to the differential equation of oscillation of a simple pendulum of length *L*,

(15) $$\frac{d^{2}\psi }{dt^{2}}+\frac{g}{L}\sin\psi =0$$

Equation (15) describes the deflection of the pendulum. Note how the spatial variable *s* in Equation (14) corresponds to time variable *t* in Equation (15). Static bending of a beam is a boundary value problem, while motion of a pendulum is an initial value problem. However, despite this difference, an analogy exists between the motion of a pendulum and the shape of a buckled elastic rod. Now, we will consider the analogy of a beam with an inverted pendulum.

Consider bending of a beam under a tensile load *F* as shown in Figure 2b. The beam is bent 360° making an approximate circle. The inset in Figure 2b shows a free-body diagram of a small section of the beam near its end. The equilibrium of the beam corresponds to the value of the bending moment *M* = *FΔy*, which is proportional to the displacement *Δy*. The expression for the bending moment can be written as EIdψds=FΔy. Here, we study whether the equilibrium of the beam is stable (straight beam) or unstable (bended beam). Differentiating the expression for the bending moment with respect to *s* and assuming $\underset{\Delta s\to 0}{\lim}\frac{\Delta y}{\Delta s}=\psi$ we obtain:

(16) $$\frac{d^{2}\psi }{ds^{2}}-\frac{F}{EI}\psi =0$$

which is similar to the equation of motion of an inverted pendulum for small angular displacement, $\frac{d^{2}\psi }{dt^{2}}-\frac{g}{L}\psi =0$. The equation for the inverted pendulum has a solution of the exponential form $\psi =c_{1}e^{\sqrt{g/L}t}+c_{2}e^{-\sqrt{g/L}t}$. The angular displacement grows exponentially with time *t* implying instability. Equation (16) for the beam has a trivial solution ψ=0, and a nontrivial solution $\psi =c_{1}e^{\sqrt{F/EI}s}+c_{2}e^{-\sqrt{F/EI}s}$. The nontrivial solution suggests that the slope of the beam grows exponentially from the point of application of the force.

On the basis of Kirchhoff’s analogy, the beam under compressive loading corresponds to the stable regular pendulum (Figure 2a), whereas the buckled beam under tensile loading corresponds to the unstable inverted pendulum (Figure 2b). An inverted pendulum can be stabilized by harmonically vibrating its foundation. Similarly, a buckled beam can be stabilized by a spatial periodicity in the geometry of the beam (Figure 2c).

If the properties of the elastic rod are changed in a periodic manner with small amplitude h<<1 and frequency Ω about the stationary value $EI_{0}$ such that:

(17) $$EI=EI_{0}\left(1+h\Omega \cos\Omega s\right)\approx \frac{EI_{0}}{\left(1-h\Omega \cos\Omega s\right)}$$

**Figure 3:**
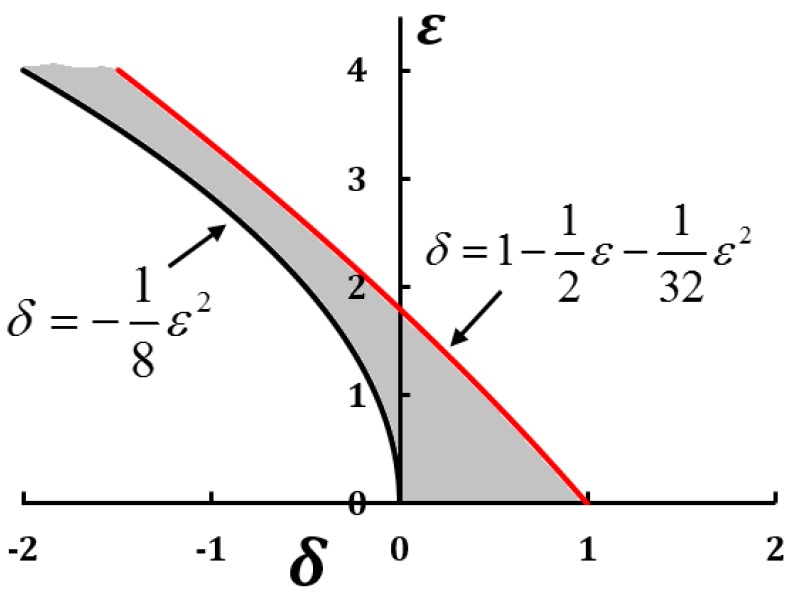
Ince–Strutt stability diagram for a beam.

Equation (16) attains the form:

(18) $$\frac{d^{2}\psi }{ds^{2}}-\frac{F}{EI_{0}}\left(1-h\Omega \cos\Omega s\right)\psi =0$$

which is similar to Equation (12) for an inverted pendulum on a harmonically vibrating foundation. Equation (18) can be converted into the canonical form of the Mathieu equation:

(19) $$\frac{d^{2}z}{d\kappa^{2}}-\left(\delta +\varepsilon \cos2\kappa \right)z=0$$

where $\kappa =\frac{\Omega s}{2},\;z\left(\kappa \right)=\psi \left(s\right),\;\delta =\frac{4F}{EI_{0}\Omega^{2}},\;\varepsilon =-\frac{4Fh}{EI_{0}\Omega }$. The stability and instability of the Mathieu equation can be studied by Lindstedt–Poincaré perturbation method [20,21]. The stability curves are obtained as:

(20) $$\delta =-\frac{1}{8}\varepsilon^{2}+\ldots ,\;\delta =1-\frac{1}{2}\varepsilon -\frac{1}{32}\varepsilon^{2}-\ldots$$

and can be represented on the Ince–Strutt stability diagram (Figure 3). The solution of Equation (19) is stable for values of (*δ*, *ε*) that lie in the shaded region between the stability curves shown in Figure 3. The negative values of *δ* is of importance in this discussion because of the tensile nature of the force *F*. For example, *F* = −4200 N, flexural rigidity $EI_{0}$ = 14 Nm^2^, h = 0.1 m, Ω = 245 m^−1^ yields the values *ε* ≈ 0.5, *δ* ≈ −0.02, which lie in shaded region. Thus, in this case the beam is stabilized by the spatial periodicity in its geometry.

The spatial periodicity of the beam can be interpreted as distributed bending moments along the beam, which can be replaced by an effective stabilizing shear force, *V*, as shown in Figure 2c. Therefore, the periodicity in the geometry of the beam manifests as an effective shear force. This shear force can stabilize the beam when the condition:

(21) $$-\frac{1}{8}\varepsilon^{2}<\delta <1-\frac{1}{2}\varepsilon -\frac{1}{32}\varepsilon^{2}$$

is satisfied. Otherwise, the beam is unstable with an exponentially increasing slope. We conclude that a pattern on the surface profile of a rod affects destabilization of the rod just as small fast vibrations affect the stability of an inverted pendulum.

## 3. Wetting and Membranes

In the preceding section, we studied how small fast vibrations or small-amplitude spatial structures can be substituted by an effective energy term, which can lead either to an effective force (such as the vibro-levitation force) or affect mechanical or phase equilibrium. In this section, we will focus on the effect of small vibrations and structures on wetting, and, specifically, on the filtration.

### 3.1. Superhydrophobicity: How Surface Patterns Change Wetting and Phase State

Non-wetting can be achieved using temporal patterns as seen in the case of vibro-levitating droplets. Oil droplets were seen to levitate indefinitely over a vibrating oil surface in the frequency range 35–350 Hz. The thin film of vapor between the droplet and the vibrating surface is stabilized by vibrations [7]. Similarly, non-wetting can be achieved on superhydrophobic surfaces with the help of micro/nano topography.

Wettability of a surface is usually characterized by the contact angle (CA), *θ*, which a droplet of liquid makes with a solid surface. On a hydrophobic surface a water droplet makes $\theta >90^{\circ }$, while on a hydrophilic surface a water droplet makes $\theta <90^{\circ }$. For an ideally smooth homogenous surface, the equilibrium CA $\left(\theta_{0}\right)$ of a liquid droplet (say, of water) is given by the Young equation

(22) $$\cos\theta_{0}=\frac{\gamma_{SA}-\gamma_{SW}}{\gamma_{WA}}$$

where $\gamma_{SA}$, $\gamma_{SW}$, and $\gamma_{WA}$ are the surface free energies of the solid-air, solid-water, and water-air interfaces. However, on real surfaces with roughness [22,23] and chemical heterogeneity, the observed CA can be different from $\theta_{0}$. In such cases, the CAs are estimated by Wenzel and Cassie–Baxter models [24,25].

**Figure 4:**
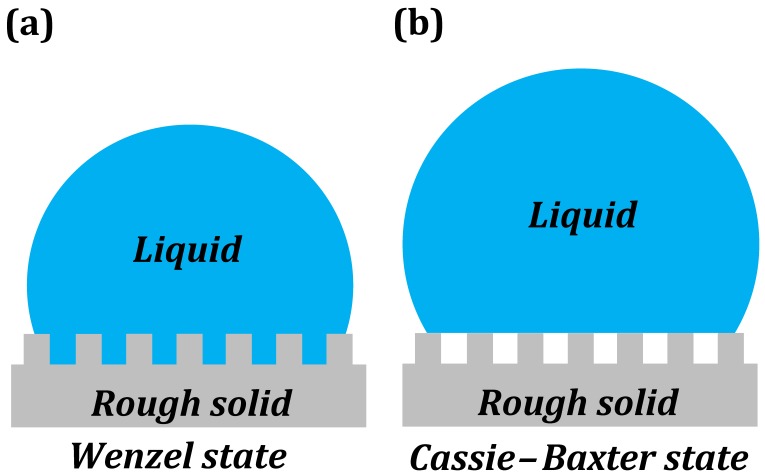
Wetting states. (**a**) A liquid droplet in Wenzel state; (**b**) A liquid droplet in Cassie–Baxter state.

The Wenzel model (Figure 4a) gives the effective CA on a rough, chemically homogenous surface.

(23) $$\cos\theta_{W}=R_{f}\cos\theta_{0}$$

where roughness factor $R_{f}\geq 1$, is the ratio of the solid surface area to the projected area. We can see from Equation (23) that roughening a hydrophobic surface makes it more hydrophobic (larger CA), while roughening a hydrophilic surface makes it more hydrophilic (lower CA). On a superhydrophilic surface, the water droplet spreads out into a thin film.

If a rough surface harbors pockets of air, thus creating chemical heterogeneities, then the CA is given by the Cassie–Baxter model (Figure 4b).

(24) $$\cos\theta_{CB}=r_{f}f_{SL}\cos\theta_{0}-1+f_{SL}$$

where *r_f_* is the roughness factor of the wet area, and 0 ≤ *f_SL_* ≤ 1 is the fractional solid-liquid interfacial area. The air pockets can lead to the surface being superhydrophobic. On superhydrophobic surfaces, water beads up into a near-spherical shape.

In both above cases, we see that surface texture (roughness) is an essential parameter in determining the wettability (or non-wettability) of a surface. On a superhydrophobic surface, a water droplet effectively “freezes” into a spherical shape. The roughness features on the superhydrophobic surface also harbor and stabilize pockets of air. On a superhydrophilic surface, a water droplet effectively “melts” into a thin film, just like the coalescence of a droplet into a liquid bath.

In order to perform averaging of surface energy, we consider a 2D system—a solid rough surface of length L along the x-axis with unit width. The roughness profile of which is given by *F(x)* whereas the local surface free energy is *γ*(*x*). The roughness factor of the surface can be written in the integral form as:

(25) $$R_{f}=\frac{1}{L}\int_{0}^{L}\sqrt{1+{\left(\frac{dF(x)}{dx}\right)}^{2}}dx$$

Similar to the averaging of small fast vibrations over time in Equation (11), the effect of surface topography and chemical heterogeneity can be incorporated into the effective surface free energies of the interface as an integral over the spatial coordinate *x*.

(26) $${\left(\gamma \right)}_{eff}=\frac{1}{L}\int_{0}^{L}\gamma \left(x\right)\sqrt{1+{\left(\frac{dF(x)}{dx}\right)}^{2}}dx$$

For a chemically homogenous rough surface, the surface energy is constant (*γ*(*x*) = constant) and Equation (26) yields the Wenzel equation, in which the surface free energy is augmented by the roughness factor *R_f_*. For a chemically heterogeneous smooth surface, Equation (26) yields the Cassie–Baxter equation. The modification of the surface free energies in Equation (26) using the average of the product of the surface free energy and the surface profile over a length is similar to the augmentation of the effective potential energy in Equation (10), with a term averaged over time. The effective surface energy and, thus, the CA can be modified by controlling the surface texture and chemistry.

**Figure 5:**
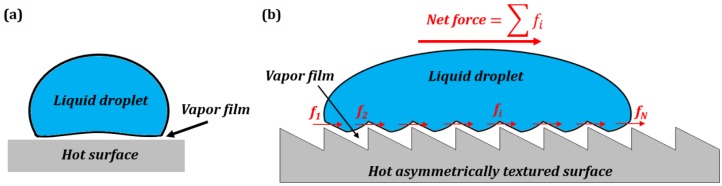
Levitating liquid droplet over a sufficiently hot surface. (**a**) Due to the Leidenfrost effect; (**b**) Self-propelled Leidenfrost droplets on an asymmetric saw-tooth surface [6].

Marmur suggested that appropriate texturing of a surface can lead to stable air films on underwater surfaces resulting in underwater superhydrophobicity [26]. Later on, Patankar and co-workers studied surface texture-induced phase transitions [14,27,28]. They investigated how surface texture affects the Leidenfrost effect [29] manifested by water droplets levitating over a sufficiently hot skillet due to the presence of an evaporating vapor film (Figure 5a). Such a film is formed only when the hot surface is above a critical temperature, whereas at lower temperatures the vapor film collapses. However, the critical temperature can be reduced, and the vapor film collapse can even be completely suppressed [30] when micro-textured superhydrophobic surfaces are used [14]. Their result demonstrated that the surface texturing can potentially be applied to control other phase transitions, such as ice or frost formation, and to the design of low-drag surfaces in which the vapor phase is stabilized in the grooves of textures without heating.

The concept was further expanded by Jones et al. [27], who showed that surface texturing can stabilize the vapor phase of water, even when liquid is the thermodynamically favorable phase. Furthermore, the reverse phenomenon exists, when patterned hydrophilic surfaces keep a liquid water layer at high temperatures when it would otherwise boil. Thus, nanoscale roughness can be applied to manipulate the phase of water. The molecular dynamics simulations demonstrated that the vapor and liquid phases of water adjacent to textured surfaces are stable. Patankar [28] has also identified the critical value of roughness, below which the vapor phase is sustainable and/or trapped gases are kept in roughness cavities or valleys, thus maintaining the immersed surface dry.

Linke et al. [31] demonstrated that surfaces with small asymmetric texture (saw-tooth profile) can induce self-propulsion in Leidenfrost droplets, and in the process, the droplets climb over the steep sides of the surface texture [32]. Leidenfrost droplets levitate on a thin film of vapor formed when the droplet contacts a surface whose temperature is much greater than the boiling point of the liquid. The vapor phase is expelled from under the droplet due to the pressure gradient in the film between the peaks and the valleys of the surface profile. Due to the inherent asymmetry of the surface texture, the vapor leaks out asymmetrically from under the droplet, causing a net directional flow of vapor. The resultant viscous forces entrain the droplet in the same direction (Figure 5b) [33,34,35]. The self-propulsion effect has potential application, such as in a sublimation heat engine [36].

The viscous force generated per tooth of the saw-tooth profile is given [32] by using momentum balance:

(27) $$f_{i}=\eta \frac{U}{h_{F}}r_{c}\lambda$$

where *η* is the viscosity of the vapor, *U* is the velocity of the vapor flow, *h_F_* is the average thickness of the vapor film, *r_c_* is the contact radius of the droplet, and *λ* is the tooth length. If there are *N* teeth below the droplet, then the net propulsion force can be obtained as:

(28) $$F=\sum_{i=1}^{N}\eta \frac{U}{h_{F}}r_{c}\lambda =\eta \frac{U}{h_{F}}r_{c}N\lambda$$

For the values *η* = 1.9 × 10^−5^ Pa s, *U* = 0.2 m s^−1^, *h_F_* = 10 μm, *r_c_* = 2.5 mm, *λ* = 1 mm, and *N* = 5, the force is *F* = 4.75 μN.

The summation of forces over an area due to surface patterns in Equation (28) is similar to the integration of small fast vibrations in Equation (11). The vibrations can be substituted by an effective stabilizing force. Similarly, the surface topography manifests as a propulsion force.

We saw how asymmetric surface patterns on a surface can be substituted by an effective force that spontaneously propels a Leidenfrost droplet over steep inclines. In general, the phenomenon of surface texture-based phase transition can be described as suppressing the boiling point and, thus, is similar to superheating or subcooling of water. Similar to the vibration-induced phase transitions, the effect of the small spatial pattern is in changing the phase state of the material.

### 3.2. Water Flow through a Vibrating Pipe with Hysteresis

**Figure 6:**
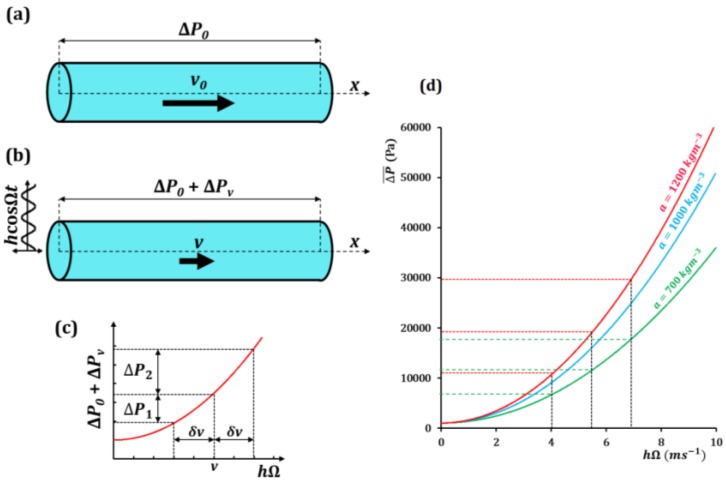
Fluid flow through a pipe. (**a**) The pressure difference Δ*P*_0_ is a nonlinear function of the flow velocity *v*_0_; (**b**) Longitudinal external vibrations *h*cosΩ*t* results in an additional pressure difference Δ*P_v_*; (**c**) Pressure difference as a function of the velocity *h*Ω. The hysteresis (Δ*P*_2_ − Δ*P*_1_) due to a small change in velocity *±δv* can significantly alter the flow characteristics [6]; (**d**) Pressure difference as a function of *h*Ω for three different fluids with *a* = 700, 1000, and 1200 kg m^−3^.

In this section, we study the effects of small fast vibrations on the flow through a hole. First, let us consider a macroscopic flow of a fluid through a pipe as shown in Figure 6a, with the mean flow velocity *v* related to the pressure loss ΔP by the nonlinear relation:

(29) $$\Delta P=av^{2}$$

where *a* is a constant. Note that for laminar flow, the dependency between the pressure and flow velocity is linear. However, in a non-ideal situations non-linearity can emerge, represented by the quadratic dependency in Equation (29), which may be a consequence of various factors, such as the turbulence, non-linear viscosity, or asymmetric variations in the pipe profile. The non-linearity is essential since it results in hysteresis [8].

To apply the averaging method, let us assume a slow velocity *v*_0_ which changes negligibly over a time period 2π/Ω. If the pipe is subjected to a fast external vibration (Figure 6b) in the form of x=hcosΩt, where *h* is a small constant amplitude, then the additional fast component of velocity is $\dot{x}=-h\Omega \sin\Omega t$. The standard assumption of the method of separation of motions is that the flow velocity is small in comparison with the amplitude of the velocity of vibrations, *h*Ω. The flow velocity can be written as the sum of the slow and fast components.

(30) $$v=v_{0}-h\Omega \sin\Omega t$$

Substituting Equation (30) into Equation (29):

(31) $$\Delta P=a{\left(v_{0}-h\Omega \sin\Omega t\right)}^{2}$$

$$\Delta P=a{v_{0}}^{2}+a{\left(h\Omega \sin\Omega t\right)}^{2}-2av_{0}h\Omega \sin\Omega t$$

(32) $$\Delta P=\Delta P_{0}+a{\left(h\Omega \sin\Omega t\right)}^{2}-2av_{0}h\Omega \sin\Omega t$$

where $\Delta P_{0}$ is the pressure loss due to *v*_0_, which changes negligibly over 2π/Ω. Averaging Equation (32) over the period 2π/Ω, similar to the temporal averaging in Equation (11) gives:

(33) $$\bar{\Delta P}=\Delta P_{0}+\frac{\Omega }{2\pi }\int_{0}^{2\pi /\Omega }a{\left(h\Omega \sin\Omega t\right)}^{2}dt$$

(34) $$\bar{\Delta P}=\Delta P_{0}+\frac{a}{2}{\left(h\Omega \right)}^{2}$$

In Equation (34), the effect of the fast vibrations is perceived as the additional pressure difference $\Delta P_{v}=a{\left(h\Omega \right)}^{2}/2$, which can intensify or weaken the fluid flow thought the pipe. Equation (34) is similar to Equation (10) in that vibrations augment the potential energy of the system, and at certain values of hΩ the vibrations can effectively stop the fluid flow. The dependency of the pressure difference in the value hΩ is shown in Figure 6c based on Equation (34). Since the dependency is non-linear, for any small change in velocity ±δv due to the external vibrations, the corresponding total change in ΔP is non-zero, as shown. The pressure difference $\Delta P_{2}$ for a small increase in velocity is greater than the pressure difference $\Delta P_{1}$ for a small decrease in velocity. This hysteresis can affect the flow in the pipe and under certain conditions even stop the flow. Using values of $\Delta P_{0}$ = 1 kPa, and *a* = 700, 1000, and 1200 kg m^−3^ (similar to the densities of gasoline, water and glycerin, respectively), a plot of Equation (34) is shown in Figure 6d. If the hydrostatic pressure driving the flow is $\Delta P_{in}$, the fluid in the vibrating pipe ceases to flow when:

(35) $$\frac{a}{2}{\left(h\Omega \right)}^{2}>\Delta P_{in}$$

Instead of a vibrating pipe, if we consider a vibrating fluid container with a hole at the bottom, the velocity of the fluid drainage is related to the static pressure head (*H*) of the fluid in the container as $v\propto \sqrt{2gH}$. Therefore, the nonlinearity in Equations (29) and (35), still holds. Thus, the drainage of fluid through the hole can be stopped by controlling the amplitude and frequency of the vibrations.

We showed how small fast vibrations can affect fluid flow through a hole, and, under certain conditions, effectively act as a valve. Next, we extend this principle to the case of vibrating membranes on the micro/nanoscale.

### 3.3. Liquid Penetration through Pores in Vibrating or Patterned Membranes

Semipermeable membranes (e.g., biological cell membranes) allow only certain molecules or ions to pass through. Osmosis is the transport of solvent molecules, such as water, through a semipermeable membrane from a region of higher to lower solvent chemical potential until the chemical potentials equilibrate. Osmosis is driven by the concentration gradient of the solute across the membrane, or, in other words, the chemical potential difference of the solvent across the membrane. The excess external pressure that must be applied to prevent the osmotic flow is called the osmotic pressure *π*. The osmotic pressure is given by the van’t Hoff equation:

(36) $$\pi =c_{solute}RT$$

where *c_solute_* is the molar concentration of the solute in the solution, *R* is the gas constant, and *T* is the absolute temperature. When an external pressure greater than the osmotic pressure *π* is applied to reverse the flux of solvent molecules then the process is called reverse osmosis (RO).

A novel principle of phase separation (e.g., water and oil from their mixture) has already been suggested using the membranes, which are hydrophilic but oleophobic, or hydrophobic but oleophilic. Note that oil, and other organic non-polar liquids, typically has a surface energy much lower than that of polar water. Because of this, hydrophilic materials are usually also oleophilic. However, dealing with the underwater oleophobicity, one can find materials which are hydrophilic but still repel oil when immersed in water.

**Figure 7:**
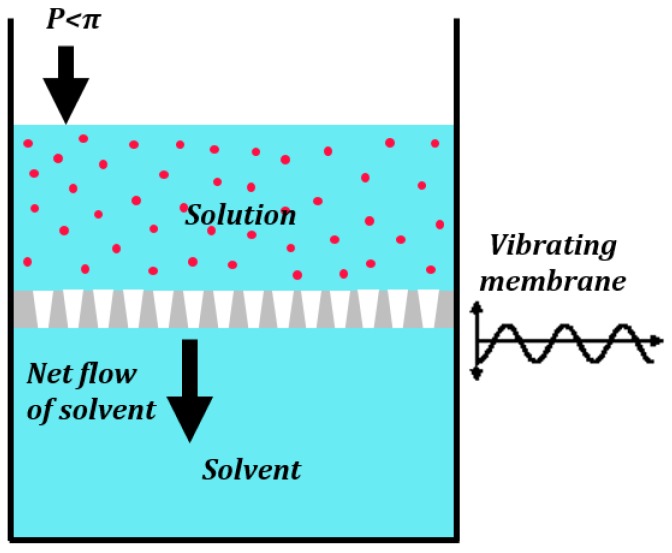
Vibrations can change the permeability of membranes. This may lead to a reverse osmotic flux even when the applied pressure is less than the osmotic pressure [6].

In the previous section, we saw how vibrations could manifest as a pressure affecting the fluid flow. For a vibrating membrane (Figure 7) consisting of several holes, the vibrations manifest as an effective pressure $a{\left(h\Omega \right)}^{2}/2$, as seen in Equation (34). The vibrations can change the effective membrane permeability if:

(37) $$\frac{a}{2}{\left(h\Omega \right)}^{2}>\pi$$

The RO process is used commonly to desalinate water. RO membranes are porous structures used in the RO process. Solvents usually take a tortuous path through the RO membranes. Note that RO is used for separation of a solvent from a solution. However, a completely different principle can be used to separate liquid mixtures using patterned superhydrophobic surfaces.

One of the recent applications of surfaces with tailored wettability is in separation of oil-water mixtures [37]. Porous media/meshes, which are selectively wetted by either water or organic solvents, can be used in this process. These porous material are analogous to the RO membranes used in desalination. The common terminology associated with wetting of a surface by oil are defined as follows. Oleophilic surfaces display oil CA less than 90°. Oleophobic (oil CA greater than 90°) surfaces used for oil-water separation need to operate in the three phase solid-oil-water system instead of the usual solid-water-air system. This calls for underwater oleophobic surfaces [38] which exhibit oil CA greater than 90° in the solid-oil-water system. Surfaces that are superhydrophobic and oleophilic, or hydrophilic and underwater oleophobic can be used to separate out oil from water.

Natural and artificial materials have been used for oil-water separation. Kapok plant fiber which is naturally hydrophobic and oleophilic was seen to separate diesel oil from water. Kapok, which is wetted by diesel due to capillary rise, can be dried and reused [39]. Artificial membranes are made by using porous/meshed structures with specific pore sizes, which may be roughened and coated with a surface agent to tailor their wetting properties. The wetting properties depend on the pore size, surface roughness and surface agent used. Stainless steel and copper meshes, and filter paper [40], were commonly used to separate mixtures in which oil is layered over water. If one of the phases in oil-water mixture is dispersed in the other as small droplets (smaller than the pore size) the meshes become ineffective. Hydrophobic porous media has been developed for separation of oil-water emulsions with and without surfactants [41,42,43,44]. Table 1 summarizes the literature which discusses various types of oil-water filtering membranes.

The rough surface of the mesh for oil-water separation having pores of radius *w* is wetted partially by oil, water and air. The effective surface free energy of a rough chemically heterogeneous surface is given by Equation (26). The solid-liquid interface area in any single pore is augmented by the factor $f_{SL}{\left(r_{f}\right)}_{L}$. The capillary pressure $P_{cap}$ across the interface of the liquid *L* is given by the force balance $P_{cap}w^{2}=2wf_{SL}{\left(r_{f}\right)}_{L}\gamma_{LV}\cos\theta_{0}$, which simplifies as:

(38) $${\left(P_{cap}\right)}_{L}=\frac{2f_{SL}{\left(r_{f}\right)}_{L}\gamma_{LV}\cos\theta_{0}}{w}$$

where $\gamma_{LV}$ is the surface free energy of the liquid-vapor interface. Note that the effect of the surface micro/nanotopography is incorporated into Equation (38) via the roughness factor. The roughness factor is the surface profile averaged over an area. This is similar to the effect of vibrations averaged using the temporal integral in Equation (11). The capillary pressure at a solid-oil interface is

(39) $${\left(P_{cap}\right)}_{oil}=\frac{2f_{oil}{\left(r_{f}\right)}_{oil}\gamma_{oil}\cos\theta_{oil}}{w}$$

whereas the capillary pressure at a solid-water interface is

(40) $${\left(P_{cap}\right)}_{water}=\frac{2f_{water}{\left(r_{f}\right)}_{water}\gamma_{water}\cos\theta_{water}}{w}$$

where $\gamma_{oil}$, $\gamma_{water}$, $\theta_{oil}$, $\theta_{water}$ are the surface free energy of the oil-vapor interface, the surface free energy of the water-vapor interface, the equilibrium CA of oil and the equilibrium CA of water, respectively.

The capillary pressure, given by Equation (38), determines if the liquid spontaneously flows through the mesh. For a hydrophobic, oleophilic mesh ${\left(P_{cap}\right)}_{water}$ is negative, whereas ${\left(P_{cap}\right)}_{oil}$ is positive and as a result, oil selectively permeates though the pores; water permeates only if an external pressure is applied to negate ${\left(P_{cap}\right)}_{water}$. For example, for a mesh of pore size *w* = 10 μm with the values *r_f_* = 2.0, *γ_water_* = 72 mNm^−1^, *θ_water_* = 107°, *f_water_* = 0.19, *γ_diesel_* = 23 mNm^−1^, *θ_diesel_* = 60°, *f_diesel_* = 1.0, we obtain ${\left(P_{cap}\right)}_{water}$ = −1.6 kPa and ${\left(P_{cap}\right)}_{diesel}$ = 4.6 kPa. The mesh will stop the water, while allowing diesel through the pores. In the case of a mesh used for oil-water separation, micro/nanotopography augments the capillary pressure and is therefore a critical factor.

Equation (35) is the critical condition for flow through a vibrating pipe or out of a vessel, while Equation (37) is the critical condition for the permeability of a vibrating membrane. The vibrations were averaged over time. In Equation (38) the surface micro/nanotopography was averaged over the area. In essence micro/nanotopography can affect the mesoscale transport through porous media while small fast vibrations can affect the molecular transport through porous media.

Note the similarity between Equation (36) for osmotic pressure and Equation (38) for the capillary pressure. Additionally, note that the osmotic pressure is independent of the membrane properties, whereas the capillary pressure for wetting is dependent on the surface characteristics. The effect discussed in this section is different from that of classical osmosis. Osmosis is a molecular scale effect and the expression for the osmotic pressure in Equation (36) is derived from thermodynamics. The pattern-induced liquid separation, which we suggest to be referred to as “pseudo-osmosis”, is a mesoscale effect with a characteristic length scale (i.e., the superhydro/oleophobic/philic surface pattern of nanometers).

## 4. Conclusions

We discussed how small fast vibrations (temporal patterns) and micro/nanotopography (spatial patterns) can affect physicochemical properties. We used Kirchhoff’s dynamical analogy, which draw parallels between spatial patterns and vibrations. We also applied Kapitza’s method of separation of motions as a tool to find an effective force that can be substituted for small fast vibrations. We applied this tool to several examples, including the flow of liquid though vibrating pipes and membranes. In all these cases, we derived an expression for an effective force that can be substituted for vibrations or patterns.

Novel biomimetic membranes which are hydrophilic but oleophobic or hydrophobic but oleophilic can be developed using this principle. The separation of oil-water mixture using selectively wettable membranes/meshes is similar to the molecular osmotic transport across a semipermeable membrane; however, the principle is different since the phenomenon is not at the molecular scale.

It is important to note that, in all the cases discussed in this paper, vibrations or surface patterns lead to some nonlinearity or hysteresis, which results in a peculiar behavior such as stabilization and propulsion. Thus, spatial and temporal patterns can affect material and surface properties. Potential applications include smart materials with tunable properties. The approach developed in our paper allows estimating system design and performance by knowing the properties of small scale vibrations and patterns. More importantly, we suggest a general method to study how small patterns affect macroscale wetting properties with superhydrophobicity and oil-water separating membranes being examples of where the method can be applied.

## Figures and Tables

**Table 1:** Summary of recent literature on oil-water separation using selectively wettable membranes.

Porous Material	Surface Texturing and Treatment	Wetting Property	Separates
Kapok plant fiber [39]	None	Hydrophobic, oleophilic	Diesel from water
Stainless steel mesh [45]	Polyacrylamide hydrogel polymerization	Superhydrophilic, underwater superoleophobic	Vegetable oil, gasoline, diesel, crude oil, n-hexane, and petroleum ether from water with 99% efficiency
Stainless steel mesh [46]	Vertically-aligned multi-walled carbon nanotubes	Superhydrophobic, superoleophilic	Diesel from water
Copper mesh [47]	Etching followed by immersion in 1-hexadecanethiol	Superhydrophobic, superoleophilic	Diesel from water
Filter paper [40]	Hydrophobic silica + polystyrene	Superhydrophobic, oleophilic	Diesel from water with 96% efficiency
Stainless steel mesh [13]	Spray coating an emulsion of PTFE, polyvinyl acetate, polyvinyl alcohol and sodium dodecylbenzenesulfonate in water	Superhydrophobic, superoleophilic	Diesel from water
Copper mesh [48]	Copper hydroxide needles grown electrochemically and coated with silane	Superhydrophobic, superoleophilic	n-hexane
Stainless steel mesh [49]	Zinc oxide nano rod coating followed by immersion in stearic acid	Superhydrophobic, superoleophilic	Toluene from water
Stainless steel mesh [50]	Hexagonal ZnO nanorods	Superhydrophobic, superoleophilic	Paraffin oil from water

PTFE: Polytetrafluoroethylene.
